# Modulation of Oxidative and ER Stress Pathways by the ADAM17 Inhibitor GW280264X in LPS-Induced Acute Liver Injury

**DOI:** 10.3390/life15121877

**Published:** 2025-12-08

**Authors:** Merve Huner Yigit, Oguzhan Okcu, Mehtap Atak, Soner Karabulut, Gökhan Yıldız, Ertugrul Yigit

**Affiliations:** 1 Department of Medical Biochemistry, Faculty of Medicine, Recep Tayyip Erdogan University, 53000 Rize, Türkiye; mehtap.atak@erdogan.edu.tr; 2 Department of Pathology, Faculty of Medicine, Recep Tayyip Erdogan University, 53000 Rize, Türkiye; oguzhan.okcu@erdogan.edu.tr; 3 Department of Medical Biology, Graduate School of Health Sciences, Karadeniz Technical University, 61080 Trabzon, Türkiye; soner_karabulut45@hotmail.com (S.K.); gokhanyildiz@ktu.edu.tr (G.Y.); 4 Department of Medical Biology, Faculty of Medicine, Karadeniz Technical University, 61080 Trabzon, Türkiye; 5 Department of Medical Biochemistry, Faculty of Medicine, Karadeniz Technical University, 61080 Trabzon, Türkiye; ertugrulyigit@ktu.edu.tr

**Keywords:** acute liver injury, ADAM17, GW280264X, lipopolysaccharide, inflammation, oxidative stress, ER stress, ferroptosis, hepatoprotection

## Abstract

Background and Objectives: ADAM17, a sheddase that regulates cytokine and receptor ectodomains, amplifies inflammatory signaling. Acute liver injury (ALI) is driven by dysregulated inflammation, accompanied by both oxidative and endoplasmic reticulum (ER) stress responses. We investigated whether pharmacological inhibition of ADAM17 with GW280264X mitigates lipopolysaccharide (LPS)-induced acute liver injury by targeting these pathways. Methods: Male C57BL/6J mice received intraperitoneal LPS (10 mg/kg). GW280264X (500 µg/kg, i.p.) was administered at one and three hours post-LPS treatment. At the fifth hour, serum and liver samples were collected to determine serum ALT/AST levels and to perform hematoxylin and eosin (H&E) staining. Inflammatory (TNF-α), oxidative (MDA, 4-HNE, Fe^2+^, GSH; NRF2/KEAP1), endoplasmic reticulum (ER) stress (GRP78, ATF6, CHOP), and ferroptosis-related (GPX4, SLC7A11) markers, along with ADAM17 protein levels, were analyzed using ELISA, colorimetric assays, and Western blotting. Results: LPS triggered hepatic injury. This was accompanied by marked elevations in TNF-α, oxidative indices (MDA, 4-HNE, Fe^2+^) and ER stress proteins (GRP78, ATF6, CHOP), together with depletion of hepatic GSH. GW280264X significantly reduced AST levels, attenuated inflammatory, oxidative, and ER stress responses, and improved hepatic histopathology. GPX4 and SLC7A11 tended to increase following treatment, but the changes did not reach statistical significance and should be interpreted cautiously due to the limited sample size (*n* = 5). Similarly, ADAM17 protein levels tended to decrease, although the change was not statistically significant. Conclusions: Pharmacological inhibition of ADAM17 with GW280264X may confer early hepatoprotection in LPS-induced ALI by attenuating inflammatory, oxidative and ER stress pathways. ADAM17 inhibition yielded partial and statistically non-significant protective effects at this early stage; therefore, these findings should be considered exploratory. Future studies with larger sample sizes and longer observation periods are warranted to confirm the durability and mechanistic basis of this response.

## 1. Introduction

Acute liver injury (ALI) is a complex and rapidly progressive syndrome characterized by an abrupt loss of hepatic function in a previously healthy liver, resulting in life-threatening metabolic derangements and multi-organ dysfunction [1]. Major etiologies include drug-induced hepatotoxicity, viral hepatitis, alcohol-related injury, and ischemia-reperfusion. The pathogenesis of ALI is characterized by dysregulated systemic inflammation and hepatocyte death [2,3]. In response to hepatotoxic injury, activation of the innate immune system triggers the release of pro-inflammatory cytokines and chemokines. When excessive and uncontrolled, this cascade amplifies systemic inflammation, contributes to extrahepatic organ dysfunction, and ultimately increases mortality [4,5,6,7,8,9]. Experimentally, lipopolysaccharide (LPS), a Gram-negative endotoxin, is widely used to model potent hepatic inflammation/injury via Toll-like receptor (TLR)-mediated cytokine release [10]. Notably, accumulating evidence implicates ferroptosis as a key contributor to sepsis-associated acute liver injury (SA-ALI), thereby amplifying hepatocellular damage and inflammatory cascades [11].

Ferroptosis, an iron-dependent, non-apoptotic form of regulated cell death, is defined by intracellular Fe^2+^ accumulation, depletion of glutathione (GSH), inactivation of glutathione peroxidase-4 (GPX4), impaired function of cystine/glutamate antiporter SLC7A11, and lipid peroxidation-driven membrane damage [12,13,14,15,16,17]. In the context of hepatic injury, elevations in labile Fe^2+^ and lipid-derived reactive oxygen species (ROS) signify ferroptotic activation and its crosstalk with oxidative and endoplasmic reticulum (ER) stress responses [17].

Proteolysis of cytokines, receptors, and adhesion molecules by a disintegrin and metalloproteinases (ADAMs)—a family of Zn^2+^-dependent type I transmembrane proteases—modulates inflammatory signaling. Among them, ADAM17 (also called TACE) is a principal sheddase that regulates key inflammatory mediators [18,19,20]. However, the development of highly selective ADAM17 inhibitors has proven challenging because of the strong structural homology among metalloproteinases. Several compounds, including GW280264X (GW), inhibit both ADAM17 and ADAM10 to varying degrees, reflecting their conserved catalytic domains and overlapping substrate preferences [21,22]. ADAM17 cleaves membrane-bound tumor necrosis factor-alpha (pro-TNF-α) to its soluble form, thereby activating nuclear factor-kappa B (NF-κB) pathways and downstream oxidative and apoptotic responses [23,24]. It also sheds receptors, such as the interleukin-6 receptor (IL-6R), and can process adhesion molecules, including vascular cell adhesion molecule-1 (VCAM-1), thereby modulating leukocyte recruitment and amplifying inflammation [25,26]. Consequently, ADAM17 has emerged as a therapeutic target in systemic inflammation. Additionally, broad-spectrum metalloprotease/TACE inhibition has been shown to attenuate TNF-α release and protect against endotoxin-induced organ injury in preclinical sepsis models [27]. GW280264X is a hydroxamate-based small-molecule metalloproteinase inhibitor structurally related to batimastat and marimastat. Its hydroxamic acid group coordinates the catalytic zinc ion within the metalloprotease active site, consistent with its dual inhibition of ADAM17 and ADAM10 [28]. GW280264X—a dual ADAM10/ADAM17 inhibitor—has been explored across inflammatory pathologies, yet its impact on ferroptosis-linked injury in ALI remains unclear. Previous genetic models of liver-specific ADAM10 and ADAM17 deletion have clarified their cooperative and opposing effects on endothelial growth factor receptor (EGFR), c-Met, and tumor necrosis factor receptor 1 (TNFR1) signaling during regeneration and fibrosis [29]. To date, the effects of dual ADAM10/17 inhibition on stress-response signaling in SA-ALI remain uncharacterized.

This study provides the first integrated assessment of oxidative stress, ER stress, and ferroptosis responses during pharmacologic inhibition of ADAM10 and ADAM17. Given the clinical burden of ALI and the mechanistic interplay among ADAM17-driven cytokine shedding, oxidative and ER stress, and ferroptosis, we hypothesized that pharmacologic inhibition of ADAM17 could mitigate LPS-induced acute liver injury. To test this hypothesis, we investigated whether the dual ADAM10/17 inhibitor GW280264X confers hepatoprotection through attenuation of inflammatory signaling, reduction of oxidative and ER stress, and modulation of ferroptosis-related pathways. To this end, we quantified biochemical and molecular indices of hepatic injury-including serum alanine transaminase (ALT) and aspartate transaminase (AST) levels, hepatic TNF-α, oxidative stress [malondialdehyde (MDA), 4-hydroxynonenal (4-HNE), Fe^2+^, GSH, and nuclear factor erythroid 2-related factor 2/Kelch Like ECH Associated Protein 1 (NRF2/KEAP1)], ER stress [glucose-regulated protein 78 (GRP78), activating transcription factor 6 (ATF6), C/EBP homologous protein (CHOP)], and ferroptosis-related (GPX4, SLC7A11) markers-alongside ADAM17 expression and histopathological findings in an acute (five hours) LPS-induced model. Whereas previous studies have primarily examined genetic ablation in chronic or regenerative contexts, this work focuses on the early endotoxemic phase, providing the first pharmacologic evaluation of how concurrent ADAM10/17 inhibition influences oxidative and ER stress signaling in SA-ALI.

## 2. Materials and Methods

### 2.1. Chemicals, Antibodies, and Kits

Phosphate-buffered saline (PBS) tablets, phosphoric acid, thiobarbituric acid, sodium carbonate, 1,3,3-tetramethoxypropane, dimethyl sulfoxide (DMSO), Triton X-100, Mayer’s hematoxylin, Tris base, Tris-HCl, glycine, and a bicinchoninic acid (BCA) protein assay kit were obtained from Sigma-Aldrich (St. Louis, MO, USA). The ADAM17 inhibitor GW280264X was purchased from Aobious (Boston, MA, USA). Primary antibodies used for Western blotting (WB) were: anti-ADAM17 (sc-390859, Santa Cruz Biotechnology, Dallas, TX, USA), anti-SLC7A11 (E-AB-17625, Elabscience, Houston, TX, USA), anti-GPX4 (E-AB-93297, Elabscience), and anti-β-actin (3700, Cell Signaling Technology, Danvers, MA, USA). The protein ladder was from Thermo Fisher Scientific (Waltham, MA, USA). Nitrocellulose membranes, enhanced chemiluminescence (ECL) substrate, and horseradish peroxidase (HRP)-conjugated secondary antibodies were from Bio-Rad Laboratories (Hercules, CA, USA). Commercial ELISA kits were used to quantify liver tissue levels of KEAP1 (Cat. No. E2198Mo, BT-Laboratory, Shanghai, China), CHOP (Cat. No. E2718Mo, BT-Laboratory), 4-HNE (Cat. No. E1293Mo, BT-Laboratory), MDA (Cat. No. E0625Mo, BT-Laboratory), NRF2 (Cat. No. E1367Mo, BT-Laboratory), TNF-α (Cat. No. E-EL-M3063, Elabscience, Houston, TX, USA), GRP78 (Cat. No. E-EL-M2696, Elabscience, Houston, TX, USA), glutathione (GSH) colorimetric assay kit (Cat No. E-EL-0026, Elabscience, Houston, TX, USA) and ATF6 (Cat. No. ELK4479, Elk Biotechnology, Wuhan, China). A ferrous ion (Fe^2+^) colorimetric assay kit (Cat. No. E-BC-K139-M, Elabscience, Houston, TX, USA) was used to assess hepatic iron levels. Unless otherwise specified, all chemicals were of analytical grade.

### 2.2. Experimental Design, Animal Treatment, and Tissue Collection

Animals were randomly assigned to the four experimental groups using a computer-generated randomization list. All experimental procedures, including biochemical measurements, ELISA, and WB analyses, were performed by investigators blinded to the group allocation in accordance with the ARRIVE guidelines [30,31]. The protocol was approved by the Animal Ethics Committee of Karadeniz Technical University Faculty of Medicine (Approval No: July 2024). A total of 20 male C57BL/6J mice (6–8 weeks old; 20 ± 4 g) were obtained from the Experimental Surgery Research Center of Karadeniz Technical University (Trabzon, Türkiye). Animals were housed at 22–23 °C under a 12-h light/dark cycle with ad libitum access to food and water.

Mice were assigned to four experimental groups (*n* = 5/group):

(1) Control: 0.9% NaCl, intraperitoneal (i.p.).
(2) LPS: a single i.p. dose of lipopolysaccharide (LPS; 10 mg/kg, Sigma-Aldrich) in 0.9% NaCl.
(3) LPS + GW280264X: Mice received LPS (10 mg/kg, i.p.), followed by GW280264X (500 µg/kg, i.p.) prepared in distilled water containing 10% DMSO. Each mouse (≈20 g) received 100 µL of solution (corresponding to ≈10 µg GW280264X) administered at the 1st and 3rd hours after LPS injection. The 10% DMSO formulation ensured complete solubility of GW280264X. No precipitation or visible injection-site irritation was observed with this vehicle [32].
(4) LPS + DMSO: LPS (10 mg/kg, i.p.) plus an equivalent volume of vehicle (10% DMSO in distilled water, i.p.) at the 1st and 3rd hours.

At five hours post-LPS, animals were anesthetized with ketamine (80 mg/kg, i.p.) and xylazine (10 mg/kg, i.p.) and sacrificed by decapitation. Blood was collected into gel-separator tubes; serum was obtained by centrifugation and stored at −80 °C. Livers were excised immediately; portions were fixed in 10% neutral-buffered formalin for histology (H&E), and the remainder snap-frozen and stored at −80 °C for biochemical and molecular analyses.

### 2.3. Tissue Homogenization and Protein Extraction

Liver tissues were homogenized using two different buffer systems, depending on the downstream assays that would be performed. For ELISA and enzymatic colorimetric measurements, samples were homogenized in 1 mL PBS containing 0.01% Triton X-100 (*v*/*v*). For Western blotting, tissues were homogenized in radioimmunoprecipitation assay (RIPA) buffer (50 mM Tris-HCl, pH 7.4; 1 mM EDTA; 1% NP-40; 0.5% sodium deoxycholate; 0.1% SDS; 150 mM NaCl; 1× protease inhibitor cocktail [EDTA-free]; 5 mM NaPPi; 20 mM NaF; 2 mM Na_3_VO_4_; 1 mM PMSF) prepared in distilled water. Homogenization was performed on ice with a mechanical homogenizer at 5000 rpm for 90 s, followed by sonication (two cycles, 15 s each, 130 W, 20 kHz; VCX500, Sonics-Vibracell, Newtown, CT, USA). Homogenates were centrifuged at 10,000× *g* for 10 min at 4 °C (Allegra 64R, Beckman Coulter, Brea, CA, USA), and the supernatants were collected. Protein concentrations were determined by BCA assay (Cat. No. BCA1, Sigma-Aldrich, St. Louis, MO, USA). Protein content was normalized before ELISA, enzymatic colorimetric assays, and Western blot analyses.

### 2.4. Enzymatic Colorimetric Measurements

Serum alanine aminotransferase (ALT) and aspartate aminotransferase (AST) were measured in the Clinical Biochemistry Laboratory of Karadeniz Technical University using an enzymatic colorimetric method on an AU5800 chemistry analyzer (Beckman Coulter, Brea, CA, USA). Hepatic Fe^2+^ levels were determined using a commercial colorimetric kit (Cat. No. E-BC-K139-M, Elabscience) according to the manufacturer’s instructions. Absorbance was read at the recommended wavelength on a microplate reader, and Fe^2+^ results were normalized to tissue protein concentration.

### 2.5. Enzyme-Linked Immunosorbent Assays (ELISA)

Liver tissue levels of KEAP1, CHOP, 4-HNE, MDA, TNF-α, GRP78, NRF2, ATF6, and GSH were quantified using commercial ELISA kits, employing sandwich or competitive formats according to the manufacturer’s specifications. Absorbance was read at 450 nm using a microplate reader (SpectraMax Paradigm, Molecular Devices, San Jose, CA, USA). Values were normalized to total protein concentration (BCA) and expressed per mg protein.

### 2.6. Western Blotting

SDS-PAGE separated equal amounts of protein from liver homogenates on 7.5–10% polyacrylamide gels under reducing, denaturing conditions and transferred to nitrocellulose membranes (100 V, 75 min). Membranes were blocked with 5% non-fat dry milk in PBS-T (PBS + 0.1% Tween-20) for 1 h at room temperature, then incubated overnight (4 °C) with primary antibodies against ADAM17 (1:1000), SLC7A11 (1:1000), GPX4 (1:750), and β-actin (1:5000) in blocking buffer. After washing, the membranes were incubated with HRP-conjugated secondary antibodies (1:5000) for 1 h at room temperature. Bands were visualized using enhanced chemiluminescence and captured with a digital imaging system. Band intensities were quantified by densitometry and normalized to β-actin.

### 2.7. Histopathology (H&E)

Liver samples were fixed in 10% neutral-buffered formalin (NBF) for 24 h and processed on an automated tissue processor. Paraffin-embedded tissues were sectioned at 4 µm, stained with hematoxylin-eosin, and examined under a light microscope (Olympus BX-51, Tokyo, Japan). A blinded pathologist scored histopathological injury. Parameters assessed included edema, congestion, hemorrhage, sinusoidal dilatation, portal inflammation, lobular inflammation, ballooning degeneration, apoptosis, necrosis, steatosis, fibrosis, and hepatocyte arrangement. Each parameter was scored 0–3 (0 = none, 1 = mild, 2 = moderate, 3 = severe), and scores were summed to obtain a total liver injury score per specimen [33,34,35,36,37].

### 2.8. Statistical Analysis

Analyses were performed in IBM SPSS Statistics v23 (IBM Corp., Armonk, NY, USA). Distributional assumptions were assessed using the Shapiro-Wilk test. For normally distributed variables, one-way ANOVA was used. If ANOVA was significant (*p* < 0.05), homoscedasticity was assessed by Levene’s test: with homogeneous variances (*p* > 0.05), Tukey HSD was applied; with heteroscedasticity (*p* < 0.05), Games-Howell was used. For non-normal variables (*p* < 0.05), group comparisons utilized the Kruskal-Wallis test, with the Mann-Whitney U test employed for pairwise post-hoc tests. Multiple testing was controlled by Bonferroni correction where applicable. Parametric data are reported as mean ± standard error of the mean (SEM); non-parametric data as median (min–max). Exact *p*-values are reported unless *p* < 0.001, which is denoted as such by convention. Graphs were generated in OriginPro 2025 (OriginLab, Northampton, MA, USA).

## 3. Results

### 3.1. GW280264X Mitigates LPS-Induced Elevations of Serum AST Levels

**Figure 1:**
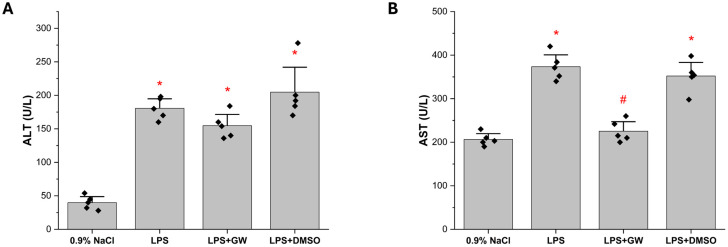
Effect of GW280264X on Serum Liver Enzymes in LPS-Induced Hepatic Injury. Serum (**A**) ALT and (**B**) AST levels. Data are presented as mean ± SEM (*n* = 5 per group). * *p* < 0.05 vs. 0.9% NaCl; # *p* < 0.05 vs. LPS group. Black squares represent individual data points for each animal.

Serum ALT and AST activities were significantly higher in the LPS group compared with the 0.9% NaCl (both *p* < 0.05). Post-LPS administration of GW280264X attenuated these elevations: AST levels were markedly reduced in the LPS + GW280264X group relative to the LPS group (*p* < 0.05), whereas ALT showed a modest but non-significant decline and remained higher than in 0.9% NaCl. The LPS + DMSO group exhibited no improvement compared with the LPS group (Figure 1).

### 3.2. Inflammatory and Oxidative Stress Biomarkers in Liver Tissue

**Figure 2:**
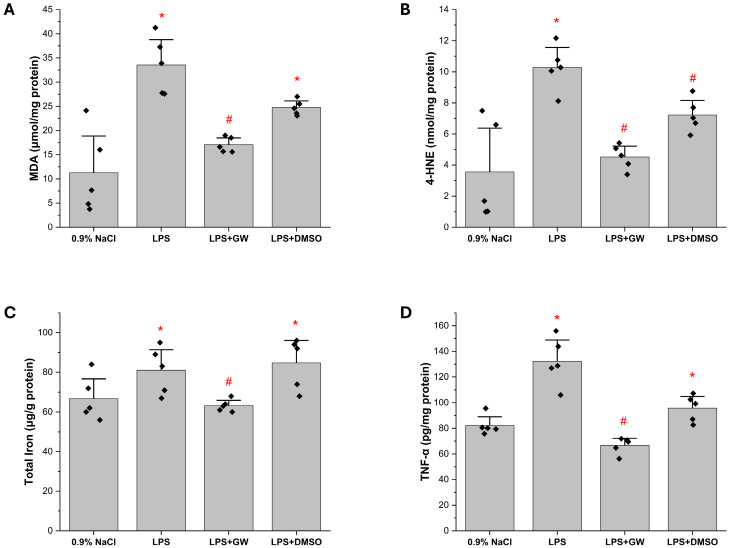
Effect of GW280264X on LPS-induced inflammatory and oxidative stress markers in liver tissue (**A**) MDA, (**B**) 4-HNE, (**C**) Iron^+^, and (**D**) TNF-α levels. Data are expressed as mean ± SEM (*n* = 5). * *p* < 0.05 vs. 0.9% NaCl, # *p* < 0.05 vs. LPS group. Black squares represent individual data points for each animal.

Hepatic TNF-α, MDA, 4-HNE, and Fe^2+^ levels were significantly elevated in the LPS group compared to the 0.9% NaCl group (all *p* < 0.05), indicating robust inflammatory activation and lipid peroxidation. Post-LPS administration of GW280264X significantly attenuated these elevations (LPS + GW280264X vs. LPS, *p* < 0.05 for each marker). The LPS + DMSO group showed no improvement and remained comparable to the LPS group across all parameters (Figure 2).

### 3.3. Redox Regulatory Markers: Keap1, Nrf2, and GSH Levels in Liver Tissue

**Figure 3:**
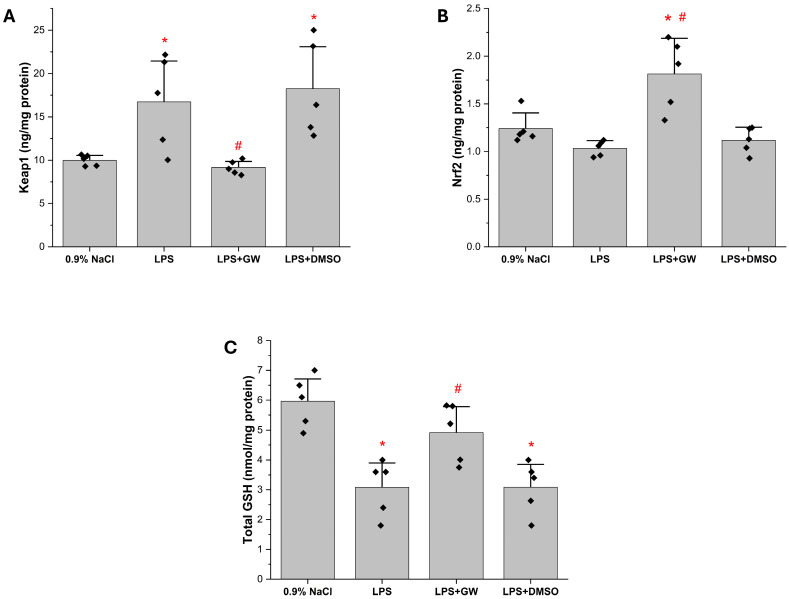
Effects of GW280264X on redox regulatory proteins in liver tissue. (**A**) Kelch-like ECH-associated protein 1 (Keap1), (**B**) nuclear factor erythroid 2 2-related factor 2 (Nrf2), and (**C**) glutathione (GSH) levels. Data are presented as mean ± SEM (*n* = 5). * *p* < 0.05 vs. 0.9% NaCl, # *p* < 0.05 vs. LPS. Black squares represent individual data points for each animal.

To probe redox regulation, hepatic KEAP1, NRF2, and total GSH levels were measured. LPS treatment significantly increased KEAP1 expression compared with the 0.9% NaCl group (*p* < 0.05); this elevation was markedly suppressed in LPS + GW280264X group (*p* < 0.05 vs. LPS). NRF2 levels were not significantly altered by LPS alone but were substantially upregulated in LPS + GW280264X (*p* < 0.05 vs. both 0.9% NaCl and LPS). Total GSH was depleted in LPS group (*p* < 0.05 vs. 0.9% NaCl) and restored following GW280264X treatment (*p* < 0.05 vs. LPS). LPS + DMSO group remained comparable to LPS across all parameters. Collectively, these findings indicate that GW280264X mitigates oxidative stress through modulation of KEAP1/NRF2/GSH (Figure 3).

### 3.4. ER Stress-Associated Protein Levels in Liver Tissue

**Figure 4:**
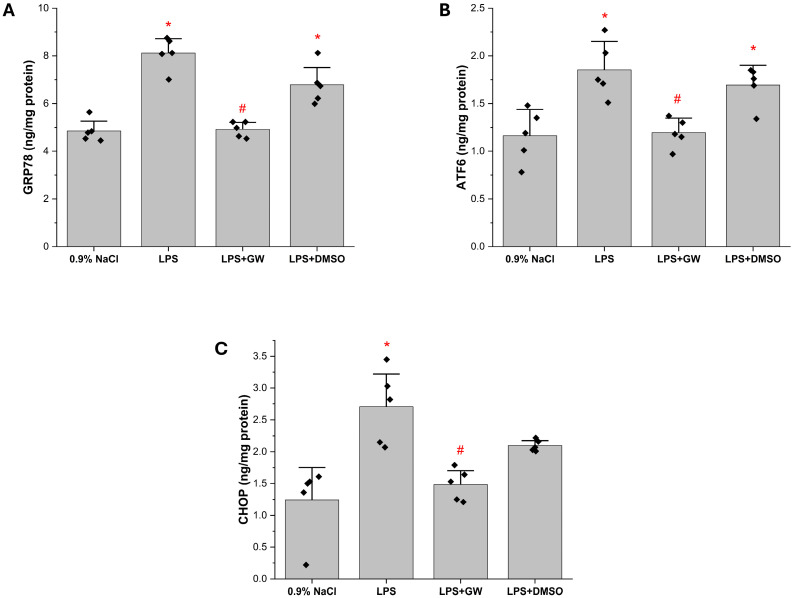
Effects of GW280264X on hepatic ER stress markers in LPS-induced acute liver injury (**A**) GRP78, (**B**) ATF6, and (**C**) CHOP levels. Data are presented as mean ± SEM (*n* = 5). * *p* < 0.05 vs. 0.9% NaCl group; # *p* < 0.05 vs. LPS group. Black squares represent individual data points for each animal.

Hepatic GRP78, ATF6, and CHOP were significantly elevated in LPS group compared with the 0.9% NaCl group (*p* < 0.05), consistent with ER stress induction. Treatment with GW280264X significantly reduced all three markers (LPS + GW vs. LPS, *p* < 0.05), indicating attenuation of LPS-induced ER stress. The LPS + DMSO group showed no significant change relative to LPS group (Figure 4A–C).

### 3.5. Protein Expression of SLC7A11, GPX4, and ADAM17

**Figure 5:**
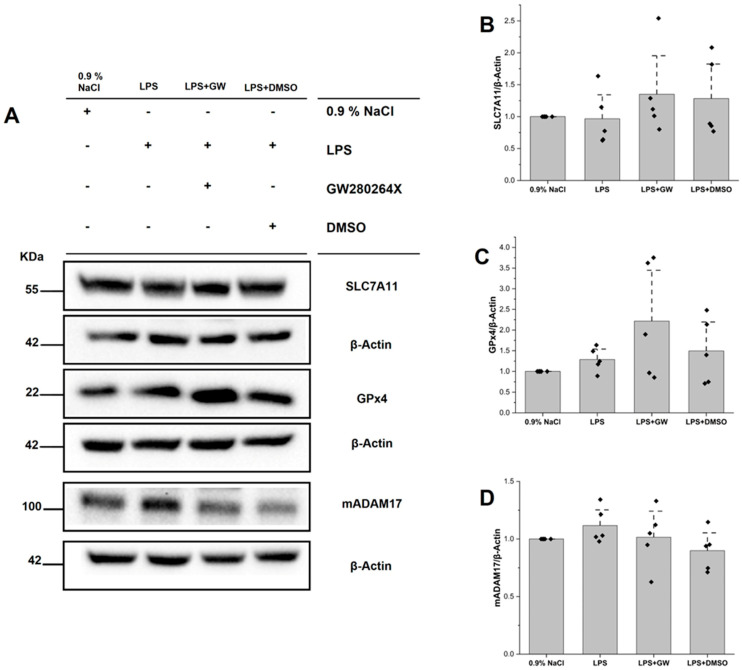
WB analysis of hepatic SLC7A11, GPX4, and mADAM17 protein expression in LPS-induced acute liver injury. (**A**) Representative WB images for SLC7A11, GPX4, mADAM17, and β-actin (loading control). (**B**–**D**) Densitometric quantification of SLC7A11 (**B**), GPX4 (**C**), and mADAM17 (**D**) normalized to β-actin. Data are presented as mean ± SEM (*n* = 5 per group). Black squares represent individual data points for each animal.

Western blotting revealed modest reductions in SLC7A11 and elevations in GPX4 in LPS-treated samples compared to 0.9% NaCl, with both proteins exhibiting upward trends after treatment with GW280264X. However, these changes were not statistically significant (*p* > 0.05). Densitometric values were normalized to β-actin. ADAM17 appeared to increase with LPS and slightly decrease in LPS + GW, but changes were likewise non-significant (*p* > 0.05). Overall, the directionality is consistent with partial preservation of ferroptosis-related defenses and putative target modulation; however, the findings are interpreted cautiously due to the limited statistical power (Figure 5A–D).

### 3.6. Liver Injury Assessment by Histology

**Figure 6:**
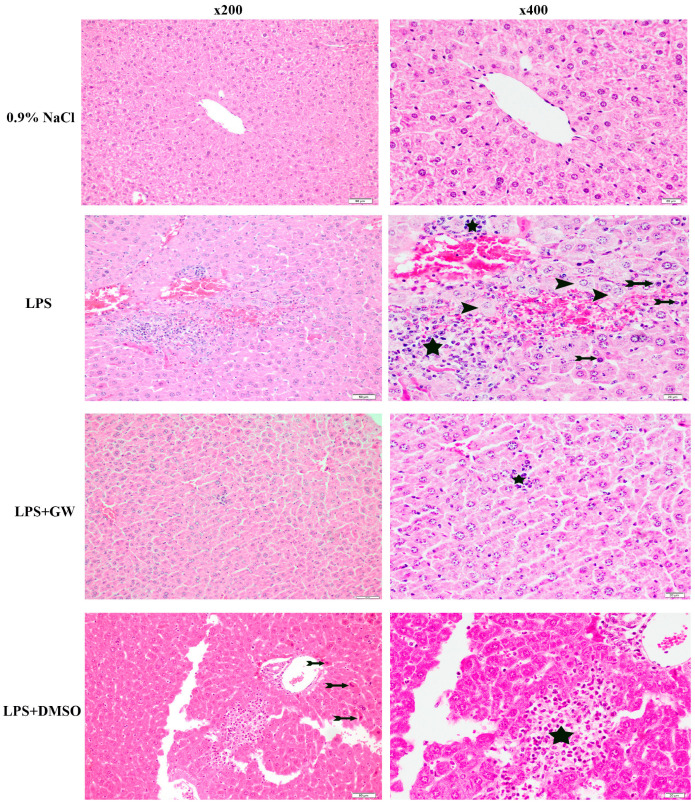
Representative photomicrographs of liver tissue sections from experimental groups stained with H&E. 0.9% NaCl group exhibits standard hepatic histological structure without signs of inflammation, cellular degeneration, or vascular alterations. LPS group exhibits extensive sinusoidal dilatation, lobular inflammation, hepatocyte swelling, and increased apoptotic activity. LPS + GW280264X group demonstrates improved tissue morphology with reduced inflammation and preservation of hepatic architecture. LPS + DMSO group displays moderate histological alterations, including mild inflammatory infiltration and sinusoidal changes. Scale bars: 50 µm and 20 µm (magnifications ×200 and ×400). Asterisks indicate inflammatory foci; arrowheads denote edema; arrows indicate apoptotic cells.

Histopathological examination revealed preserved hepatic architecture in the 0.9% NaCl group, with no evident lesions. In contrast, LPS administration induced prominent histological alterations, including sinusoidal dilatation, lobular inflammation, edema, and increased hepatocellular apoptosis. Consistent with biochemical findings, GW280264X treatment attenuated these lesions, showing less pronounced lobular inflammationcompared with the LPS group, indicating partial histological improvement. LPS + DMSO treatment group exhibited mild-to-moderate inflammatory infiltration and sinusoidal changes, remaining largely comparable to LPS group without meaningful amelioration. While a slight improvement in specific histological parameters was noted, it did not reach biological relevance. In contrast, GW280264X produced a more substantial reduction in lobular inflammation compared with both LPS alone and LPS + DMSO, suggesting a specific pharmacological effect. No hepatocyte disorganization, steatosis, or fibrosis was observed in any group. Quantitative scoring (0–3 per parameter) was combined into a total liver injury score (Table 1) and illustrated in representative micrographs (Figure 6).

## 4. Discussion

**Figure 7:**
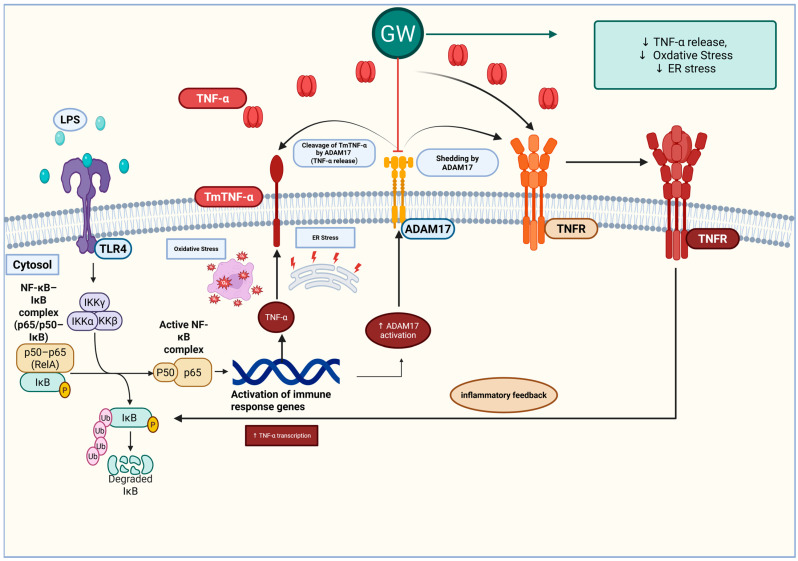
Proposed schematic illustration summarizing the putative early-phase mechanism by which GW280264X modulates TNF-α shedding and stress-related signaling pathways in LPS-induced acute liver injury. Downward arrows indicate a decrease in TNF-α release, oxidative stress, and ER stress. Created with BioRender. Huner Yigit, M. (2025) https://BioRender.com/yfwgfs4.

In a short-term LPS model of ALI, the dual ADAM17 inhibitor GW280264X conferred hepatoprotective effects. Serum AST elevations were attenuated, histological analysis revealed reduced inflammatory injury, and hepatic stress responses were broadly suppressed—including TNF-α, lipid peroxidation (MDA, 4-HNE), Fe^2+^ accumulation, depletion of GSH, and ER stress markers (GRP78, ATF6, CHOP) (Figure 7). Western blotting analysis showed directionally favorable but non-significant changes in SLC7A11/GPX4 and ADAM17 expression, suggesting limited statistical power at the 5-h time point and potential indirect modulation of ferroptotic defenses.

We acknowledge that the small sample size (*n* = 5 per group) limits the statistical power, particularly for protein-level data derived from Western blot analyses. Although the observed trends for GPX4, SLC7A11, and ADAM17 were directionally consistent with the anticipated effects of ADAM17 inhibition, none reached statistical significance. Therefore, we refrain from making definitive mechanistic claims based solely on these findings and interpret them as suggestive rather than conclusive.

Excessive TNF-α-driven inflammation is a hallmark of endotoxin-mediated liver injury. ADAM17 serves as the principal sheddase responsible for releasing soluble TNF-α and regulating downstream NF-κB signaling [23,24]. Genetic attenuation of ADAM17 in myeloid cells or hepatocytes reduces circulating TNF-α levels and improves survival following LPS challenge [38,39], while small-molecule ADAM17 inhibitors have demonstrated anti-inflammatory effects in sepsis models [27]. In our study, reduced transaminase levels and improved histological features with GW280264X treatment are consistent with attenuated cytokine-mediated injury. Targeting ADAM17 in this context may disrupt the inflammatory amplification loop associated with TNF-α. Mechanistically, GW280264X inhibits ADAM10/17 by chelating the catalytic zinc ion within the metalloprotease active site, thereby preventing proteolytic shedding of TNF-α and other cytokine receptors. The hydroxamate functional group of GW280264X coordinates the catalytic zinc ion to form a reversible complex that blocks proteolytic activity, while its biphenyl-sulfonamide moiety confers substrate affinity and dual selectivity toward ADAM10 and ADAM17 [28]. These structural features collectively account for the compound’s regulatory effects and resultant hepatoprotective outcome.

LPS challenge induced oxidative damage consistent with ferroptotic stress—elevated Fe^2+^, MDA, and 4-HNE levels accompanied by GSH depletion—which was reversed by GW280264X treatment. Although GPX4 and SLC7A11 expression changes did not reach statistical significance, their upward trends following treatment are biologically coherent, given the restoration of GSH and reduced lipid peroxidation. Previous studies have shown that mitigating ferroptosis confers protection against sepsis-associated liver injury [40,41,42,43]. Our findings suggest that ADAM17 inhibition attenuates ferroptosis indirectly—primarily through suppression of inflammation-driven ROS generation—rather than by directly upregulating GPX4 or SLC7A11.

LPS exposure increased GRP78 levels, reflecting ER stress activation, while GW280264X attenuated these responses. This finding is consistent with our previous reports demonstrating GRP78 suppression by GW280264X in lung and kidney tissues [44,45]. Crosstalk among inflammatory, oxidative, and ER stress pathways creates a self-perpetuating cycle that amplifies hepatic injury in ALI [46]. By curbing upstream cytokine signaling and oxidative burden, ADAM17 inhibition may secondarily restore ER homeostasis. Reports demonstrating that ADAM17 downregulation alleviates ER stress-related injury in other tissues support this inference [47]. Despite the reduction in CHOP levels, histological apoptosis did not show a statistically significant decrease. 

The validity of the LPS-induced acute liver injury model used in this study was confirmed by characteristic biochemical and histopathological changes, including marked elevations in serum ALT and AST levels, as well as lobular inflammation, sinusoidal dilatation, and hepatocellular apoptosis. These findings are consistent with previously established criteria for successful model induction [48,49,50]. Mechanistically, LPS activates hepatocytes via TLR4 signaling, leading to the release of TNF-α and IL-1β and subsequent oxidative and endothelial injury. The concordance between our results and these well-defined pathological features supports the robustness and reproducibility of the experimental model employed in this study.

It is important to recognize that GW280264X inhibits both ADAM17 and ADAM10, the latter of which also mediates the proteolytic shedding of several membrane proteins, including Notch, the IL-6 receptor, and cadherins. While ADAM17 is regarded as the principal sheddase of TNF-α, ADAM10 plays pivotal roles in tissue remodeling, cell adhesion, and epithelial integrity, and may influence hepatic repair or fibrosis under certain conditions. The interplay and potential compensatory mechanisms between ADAM10 and ADAM17 remain an active area of investigation, particularly when contrasting acute versus chronic liver injury models. As highlighted by Costa et al., metalloproteinases—including ADAM family members—have multifaceted roles extending beyond inflammation to encompass broader physiological and developmental processes [51]. Thus, situating ADAM17 within its protease family underscores both its therapeutic relevance and the need to avoid oversimplified single-target strategies without considering system-wide consequences. Previous genetic studies suggest that ADAM10 and ADAM17 regulate overlapping yet counterbalancing signaling networks within the liver. For instance, hepatocyte-specific deletion of either protease can disrupt EGFR, c-Met, and TNFR1 signaling, whereas their combined deficiency may partially restore equilibrium among these pathways [29]. Based on these findings, selective inhibition of one enzyme may exaggerate compensatory activity of the other, leading to excessive or misdirected cytokine and growth factor release. In contrast, dual pharmacologic inhibition with GW280264X could simultaneously modulate both proteases, reestablishing a more balanced shedding profile and potentially mitigating inflammatory amplification as well as secondary oxidative and ER stress injury. Although our current data do not directly confirm this mechanism, the observed biochemical trends and reduction in stress markers are consistent with such a re-equilibration effect.

Several metalloproteinase inhibitors have been evaluated in experimental models of acute or subacute liver injury, providing valuable context for the present findings. The selective MMP/TACE inhibitor GI129471 significantly reduced serum TNF-α levels, transaminase activity, and centrilobular necrosis in endotoxemic mice, indicating that suppression of TNF-α release is a key hepatoprotective mechanism during acute inflammation [27]. Similarly, the MMP inhibitor CTS-1027 attenuated hepatocyte apoptosis and fibrogenic activation in bile-duct-ligated mice, supporting a protective role for pharmacologic MMP inhibition in cholestatic injury [52]. In contrast, the broad-spectrum MMP/TACE inhibitor Marimastat reduced necroinflammatory injury in the acute CCl_4_ model but promoted collagen accumulation under chronic exposure, underscoring the dual role of metalloproteinases in both injury and matrix remodeling [53]. Collectively, these studies demonstrate that metalloproteinase inhibition can effectively mitigate early inflammatory and apoptotic liver damage, particularly when TNF-α signaling predominates. However, prolonged or non-selective inhibition may interfere with fibrolytic remodeling. In this context, our results showing that dual ADAM10/17 blockade alleviates LPS-induced hepatic injury are consistent with the acute hepatoprotective profile observed with selective metalloproteinase inhibition.

The strengths of this study include its multimodal experimental design—integrating biochemical, ELISA, Western blotting, and histological assessments—and the convergence of evidence across multiple stress-response pathways. Key limitations include the small sample size (*n* = 5 per group), a single early time point (5 h), partial biochemical reversal, and non-significant Western blot changes in SLC7A11, GPX4, and ADAM17 expression. Because GW280264X is a dual ADAM10/17 inhibitor, potential off-target contributions via ADAM10 cannot be excluded. Cytokine profiling beyond TNF-α (e.g., IL-6, sTNFR) and time-course kinetic analyses were not performed. Importantly, the current study captures only the early phase of LPS-induced injury, limiting insight into the persistence or resolution of hepatoprotective effects. Future studies should therefore incorporate extended observation periods (e.g., 24 h and 48 h) to better model clinically relevant progression and the durability of therapeutic responses. Finally, the present model employed LPS alone, rather than the combined LPS/D-GalN paradigm, which may activate distinct hepatocellular death pathways; thus, generalization to broader ALI etiologies should be made cautiously.

These findings provide preliminary evidence that ADAM17 represents a potential therapeutic target in ALI, with effects extending across inflammatory, oxidative/ferroptotic, and ER stress pathways. However, given the limited sample size and corresponding statistical power, the results should be interpreted with caution. Future studies employing higher-powered experimental designs are needed to confirm these mechanistic relationships and strengthen causal inference. Such investigations should (i) extend observation periods to capture both the peak and resolution phases of injury, (ii) increase statistical power for Western blot endpoints, (iii) interrogate upstream cytokine networks and downstream cell-death effectors, and (iv) evaluate GW280264X in complementary models (e.g., LPS/D-GalN, ischemia–reperfusion). Mechanistic studies incorporating cell-type-specific ADAM17 modulation and ferroptosis-targeted interventions will be critical to delineate the direct versus indirect contributions of these pathways.

## 5. Conclusions

This study demonstrates that GW280264X confers early and multi-axial, albeit limited, protection against LPS-induced acute hepatic injury. The findings suggest that pharmacologic targeting of ADAM17 warrants further evaluation in preclinical studies. By dampening upstream TNF-α signaling, GW280264X reduced ROS- and cytokine-mediated injury, accompanied by lower lipid peroxidation (MDA and 4-HNE), preservation of GSH, and attenuation of ER stress markers. These biochemical effects were consistent with ameliorated histology and partial normalization of transaminases, indicating preservation of hepatocellular integrity. Given the wide substrate repertoire of ADAM17—including factors involved in regeneration—excessive inhibition may carry risks; however, in the context of acute endotoxemia, the overall effect was protective. Future studies should validate these findings in complementary ALI models, refine dosing and timing, and optimize the balance between therapeutic efficacy and physiological repair. Collectively, these data support the concept that ADAM17 inhibition may mitigate the inflammatory–oxidative storm characteristic in ALI, representing a promising, though preliminary, strategy for severe inflammatory liver injury.

## Figures and Tables

**Table 1:** Histological Damage Scores in Liver Tissue.

Parameters Median (Min–Max)	0.9% NaCl	LPS	LPS + GW280264X	LPS + DMSO	Kruskal-Wallis, *p*
**Hepatocyte disorganization**	0 (0–0)	0 (0–0)	0 (0–0)	0 (0–0)	1
**Edema**	1 (1–2)	2 (2–3) *	2 (2–2)	1 (1–1)	*0.040173*
**Congestion**	1 (1–2)	2 (2–2)	2 (2–2)	1 (1–2)	0.138639
**Hemorrhage**	1 (0–1)	1 (1–2)	1 (0–2)	1 (0–1)	0.356239
**Sinusoidal dilatation**	1 (1–1)	2 (2–3) *	2 (2–2) *	1 (1–1)	*0.015104*
**Portal inflammation**	0 (0–0)	1 (0–1)	1 (0–1)	1 (0–1)	0.299781
**Lobular inflammation**	1 (1–1)	3 (2–3) *	1 (1–1) ^#^	2 (2–3) *	*0.019315*
**Ballooning degeneration**	1 (1–1)	1 (1–1)	1 (1–1)	1 (1–1)	1
**Apoptosis**	1 (1–1)	2 (2–2) *	2 (1–2)	1 (1–1)	*0.036971*
**Necrosis**	0 (0–0)	1 (0–1)	0 (0–0)	1 (0–1)	0.138639
**Steatosis**	0 (0–0)	0 (0–0)	0 (0–0)	0 (0–0)	1
**Fibrosis**	0 (0–0)	0 (0–0)	0 (0–0)	0 (0–0)	1
**Total Score**	7 (7–8)	16 (14–16) *	12 (9–13) *^,#^	9 (9–11) *	*0.01422*

*p* < 0.05 compared to 0.9% NaCl group (*) and LPS group (^#^) (Kruskal-Wallis test followed by Bonferroni-adjusted Mann-Whitney U tests.

## Data Availability

This published article includes all data generated or analyzed during this study.
